# Carrying Police Load Increases Gait Asymmetry in Ground Reaction Forces and Plantar Pressures Beneath Different Foot Regions in a Large Sample of Police Recruits

**DOI:** 10.3390/bioengineering11090895

**Published:** 2024-09-05

**Authors:** Mario Kasović, Andro Štefan, Lovro Štefan

**Affiliations:** 1 Department of General and Applied Kinesiology, Faculty of Kinesiology, University of Zagreb, 10000 Zagreb, Croatia; mario.kasovic@kif.hr (M.K.); andro.stefan95@gmail.com (A.Š.); 2 Department of Physical Activities and Health Sciences, Faculty of Sports Studies, Masaryk University, 625 00 Brno, Czech Republic; 3 Department of Physical Education and Sport, Faculty of Education, Catholic University in Ružomberok, 034 01 Ružomberok, Slovakia

**Keywords:** special populations, police equipment, load carriage, symmetry, effect size

## Abstract

Background: Although carrying external load has negative effects on gait biomechanics, little evidence has been provided regarding its impact on body asymmetry. The main purpose of the present study was to examine, whether standardized equipment produced greater gait asymmetries in ground reaction force and plantar pressure. Methods: For the purpose of this study, we recruited 845 police recruits (609 men and 236 women; 72.1% men and 27.9% women) measured in two conditions: (i) ‘no load’ and (ii) ‘a 3.5 kg load’. Absolute values in ground reaction forces and plantar pressures beneath the different foot regions were assessed with pedobarographic platform (Zebris FDM). Asymmetry was calculated as (x_right_ − x_left_)/0.5 × (x_right_ + x_left_) × 100%, where ‘x’ represented a given parameter being calculated and a value closer to 0 denoted greater symmetry. Results: Significant differences in ground reaction forces and plantar pressures between the left and right foot were observed, when adding ‘a 3.5 kg load’. Compared to the ‘no load’ condition, carrying ‘a 3.5 kg load’ significantly increased gait asymmetries for maximal ground reaction forces beneath the forefoot (ES = 0.29), midfoot (ES = 0.20) and hindfoot (ES = 0.19) regions of the foot. For maximal plantar pressures, only the asymmetry beneath the midfoot region of the foot significantly increased (ES = 0.19). Conclusions: Findings of this study indicate that ‘a 3.5 kg load’ significantly increases ground reaction force and plantar pressure gait asymmetries beneath the forefoot and midfoot regions, compared to ‘no load’ condition. Due to higher loads, increases in kinetic gait asymmetries may have negative effects on future pain and discomfort in the foot area, possibly causing stress fractures and deviated gait biomechanics in police recruits.

## 1. Introduction

Load carriage in special populations, like police officers, is considered a crucial component of everyday physical activity and successful performance of occupational tasks [1,2] Although such load may have beneficial effects for on-duty protection and completing the tasks at maximal level [3,4], previous research has highlighted negative effects of the load on one’s health and well-being (Salvendy, 2012), primarily focusing on physiological [3,5] and biomechanical changes [3,4]. In the field of physiology, carrying an external load and gait propulsion may produce higher energy expenditure [3]. On the other hand, from a biomechanical point of view, added mass to body weight may increase moments in the trunk, hip and knee flexion and extension areas, while inconclusive data between the load carriage and ground reaction forces and plantar pressures are detected [4]. Both physiological and biomechanical consequences of carrying heavy loads can also increase the fatigue [6] and the incidence of musculoskeletal injuries [7,8].

Studying gait symmetry has often been a topic of interest for healthcare professionals to detect gait characteristics in normal population [9] and identifying injury risk [10]. A ‘perfect symmetry’, indicating an equal degree for a given parameter between the left and right foot, has been set to be between 5 and 15% for some motor abilities, like strength [11]; it is reasonable to expect that carrying loads may naturally affect gait asymmetry for up to 50% [12]. With that in line, available studies have shown that carrying an extra load potentially increases hip and knee extensor moments of the unloaded leg [13], changing knee biomechanics [14,15]. Although a common method for assessing the degree of symmetry has been by measuring ground reaction forces [12], the majority of previous studies have investigated the effects of external load on ground reaction force and plantar pressure gait asymmetries during quiet stance [16,17], with limited information for such a phenomenon during gait [12]. A study by Zhang et al. [12] has concluded that carrying a load of 20% body mass increases ground reaction force asymmetries in a mediolateral plane, compared to 0 to 10% conditions. Indeed, an increased gait asymmetry and body compensations following load carriage come from inertial characteristics of the musculoskeletal system [18].

To the best of the authors’ knowledge, there has been a lack of studies examining the effects of load carriage on gait asymmetries in terms of ground reaction forces and plantar pressures. Since ‘a perfect symmetry’ between the sides of the body without carrying a load does not exist, we can speculate that an additional mass added onto the body may even increase gait asymmetry in the aforementioned gait parameters. Indeed, a higher level of ground reaction forces and plantar pressures have been constantly associated with a higher incidence of musculoskeletal injury rate [19,20], which can increase and prolong hospitalization time [21]. Although a load carriage is a necessity for special populations, re-distributing load items on the body may be a crucial part for minimizing negative impacts on gait biomechanics.

Therefore, the main purpose of the present study was to examine whether standardized equipment produced greater ground reaction force and plantar pressure gait asymmetries in a large sample of police recruits. We speculated that such loads might increase ground reaction force and plantar pressure asymmetries, especially beneath the hindfoot and forefoot regions.

## 2. Materials and Methods

### 2.1. Study Participants

For the purpose of this study, we recruited 900 police recruits, who were part of the Croatian police service at the time. More detailed information about recruitment, sample size characteristics, inclusion and exclusion criteria and data regulations can be found elsewhere [22]. In brief, every year, a police academy recruits and welcomes around 900 healthy men and women, who undertake a special police training program in the duration of one year. In 2023, we were able to recruit all 900 first-year police recruits for our study and 845 of them had eligible data for further analyses (27.9% women). The inclusion criteria included from all participants to be without locomotor and mental acute or chronic diseases, which could prevent them from taking part in the study, and to be in the training program of the police academy on a regular basis. The exclusion criteria included participants suffering from locomotor (injury) or mental (depression or any other disease) and who were ill at the time the study had been conducted. Before the study had been conducted, all participants became familiar with aims, hypotheses, benefits and potential risks of the study and how the findings might translate into practice. Following the Declaration of Helsinki procedure, all analyses were anonymous and all participants gave a written informed consent to participate in the study. This study was approved by the Ministry of Internal Affairs and police academy ‘Josip Jović’ and the Ethical Committee of the Faculty of Kinesiology, University of Zagreb, Croatia (ethical code number: 511-01-128-23-1).

### 2.2. Load Equipment

A standardized load equipment being carried by police recruits involved a belt with a gun and a full handgun’s magazine, an additional full handgun’s magazine, a nightstick and handcuffs, where the final weight was around 3.5 kg (7.7 Ibs) [22,23].

### 2.3. Ground Reaction Forces and Plantar Pressures

Ground reaction forces and plantar pressures beneath different foot regions were analyzed via an objective method of the Zebris pedobarographic platform (FDM; GmbH, Munich, Germany; number of sensors: 11.264; sampling rate: 100 Hz; sensor area: 149 cm × 54.2 cm). The device uses a multisensory principle which may capture spatiotemporal and kinetic gait characteristics during walking or standing positions. More detailed information of testing the protocols and generating the data can be found elsewhere [22,23]. In brief, each participant walked at a preferred gait speed over the platform eight times, after being instructed not to target the platform or change the patterns of the walk. After completing the first task, the same task was repeated while carrying police equipment. The kinetic gait parameters included generating the data regarding maximal ground reaction forces (N) and plantar pressures (N/cm^2^) of the left and right foot of the body for the forefoot, midfoot and hindfoot regions.

### 2.4. Data Analysis

All procedures were analyzed using Statistical Packages for Social Sciences software version 23 (SPSS Inc., Chicago, IL, USA). First, to test the normality of the study variables, we used the Kolmogorov–Smirnov test. For normally and not normally distributed variables, descriptive statistics are presented as mean and standard deviation (SD) or median and interquartile range (25th and 75th percentile). Student *t*-test for dependent samples or Wilcoxon singed-rank test were used to examine differences between ‘no load’ vs. ‘a 3.5 kg load’. To examine gait asymmetries, we used the formula proposed by Robinson et al. [24]: (x_right −_ x_left_)/0.5 × (x_right_ + x_left_) × 100%, where an ‘x’ represented a given parameter. A final score closer to 0 denotes a more symmetrical gait, while a score that deviates more from 0 denotes greater asymmetry. Effect size (ES) was used to express the magnitude of the difference between groups and was presented as ‘small’ (0.2), ‘moderate’ (0.5), ‘large’ (0.8) [25]. To test, whether gender had any effects on kinetic gait changes, we used repeated-measures ANOVA with gender as a between-group factor and found no significant interaction between time and gender in any of the studied variables, so we omitted sex-specific presentation of the data. Also, age and body mass index were not significantly correlated to ground reaction force and plantar pressure changes. The significance was set at a priori *p* ≤ 0.05.

## 3. Results

Socio-demographic characteristics of the study participants are presented in Table 1. The data of Table 1 relied on one previous study published by the same authors [22].

Changes in ground reaction forces and plantar pressures are presented in Table 2. When carrying ‘a 3.5 kg load’, significant differences in ground reaction forces for left forefoot (∆ = 2.9%), left midfoot (∆ = 3.6%), right midfoot (∆ = 3.6%) and right hindfoot (∆ = 1.7%) were observed. For plantar pressures, a load of 3.5 kg significantly increased the area beneath the left forefoot (∆ = 2.0%), right forefoot (∆ = 1.2%) and right midfoot (∆ = 0.4%). Finally, the % of time maximal force during stance time was significantly increased beneath the left forefoot (∆ = 0.5%) and right midfoot (∆ = 0.8%), while a significant decrease in left midfoot was shown. Changes in gait asymmetries according to gender showed no significant time* gender interactions for ground reaction forces beneath the forefoot (*F*_1,833_ = 0.616, *p* = 0.433), midfoot (*F*_1,833_ = 0.347, *p* = 0.556) and hindfoot (*F*_1,833_ = 0.750, *p* = 0.387) regions of the foot. Also, when force was applied to a surface as a plantar pressure, we observed no significant time* gender interaction for asymmetries beneath the forefoot (*F*_1,833_ = 0.743, *p* = 0.392), midfoot (*F*_1,833_ = 0.422, *p* = 0.588) and hindfoot (*F*_1,833_ = 0.255, *p* = 0.650) regions of the foot.

Table 3 shows asymmetry characteristics in ‘no load’ and ‘a 3.5 kg load’ conditions between the left and right foot. Most notably, ‘a 3.5 kg load’ significantly increased asymmetries in forefoot (ES = 0.29), midfoot (ES = 0.20) and hindfoot (ES = 0.19) regions of the foot for ground reaction forces. For plantar pressures, only the asymmetry beneath the midfoot region of the foot significantly increased (ES = 0.19). Also, the % of time maximal force during stance time significantly increased beneath the hindfoot (ES = 0.17) region of the foot, while other asymmetries were non-significant.

## 4. Discussion

This study aimed to investigate the effects of carrying load on ground reaction force and plantar pressure gait asymmetries. The main findings of this study are that (i) ‘a 3.5 kg load’ significantly increases asymmetries in ground reaction forces, especially in the forefoot and midfoot regions, and (ii) the asymmetry index in plantar pressure also increases, with the largest magnitudes being observed for the forefoot region.

Based on the findings available, this research represents one of the initial examinations of differences in asymmetry under various load conditions among police recruits. As discussed in the Introduction section, previous approaches to defining gait asymmetry between the left and right foot have typically involved measuring ground reaction forces during stance [16,26]. However, there has been limited study on asymmetrical gait analysis during actual gait [12]. Notably, when carrying heavy loads, gait asymmetry in ground reaction forces becomes more pronounced, resulting in differing impacts on the left and right foot. Previous studies have employed asymmetric/unilateral loads to assess the effects of such equipment on kinematic and kinetic gait parameters [12,13,14,15,27]. In cases of asymmetric lifting, greater loads are placed on the musculoskeletal system, particularly the trunk, when compared to symmetric lifting techniques [13]. Additionally, the increased asymmetry in ground reaction forces and plantar pressures observed in this study could be attributed to cumulative effects resulting from changes in the inertial patterns of the musculoskeletal system and the restriction of natural arm swing due to load characteristics and lateral trunk position [18]. These findings align with previous research suggesting that deviations in trunk movement away from the loaded side are indicative of motor control actions related to load carriage strategies and characteristics such as weight and shape [12]. Furthermore, it has been observed that compensations between the sides of the body are associated with preferred handedness and alterations in the neuromuscular system. In a study by Alamoudi et al. [27], 20 males carried a load of 10 lbs (≈4.5 kg) in four different modalities of frontal, lateral, bilateral and posterior positions while walking over a Kisler platform (FDM; GmbH, Munich, Germany). Similar to our findings, the compression and shear forces significantly increased with the magnitude of the weight carried, especially in lateral position. This is not surprising, since in our study, a gun with a full handgun’s magazine was positioned sideways (left or right side of the body) and might have led to even greater asymmetries. Because of the nature of the load carried, the participants counterbalanced the weight by flexing the trunk, which may have led to an increased distance between the center of mass of the body and weight [28]. Although the latero-flexion of the trunk in the opposite direction prevents from falling and restores body balance, it reduces gait stability [29] and increases gait asymmetry [12]. Also, greater gait asymmetries are often explained by the increased cadence, which occurs to reduce the stress on the joints of the lower limb [30]. Through an exploration of various factors such as load patterns and physiological adaptations [1], policymakers in the healthcare field could potentially revamp existing load structures and adjust their placement on the body. According to a study, the introduction of a ‘3.5 kg load’ was found to have a minor yet noteworthy impact on kinetic gait asymmetry. These alterations were believed to be linked to load placement [31] and increased energy consumption [32].This has been supported previously, where larger individuals classified as ‘obese’ increase their oxygen and carbon oxide consumption, relative energy expenditure and heart rate [33]. Indeed, obese individuals tend to have higher cardiac stroke volume and a higher mechanical demand on the lungs, which increase inspiratory and expiratory gas volumes and lead to breathing inefficiency [33]. To overcome this problem, we tested the interaction effect of body mass index on gait asymmetries and found non-significant main effects for both men and women, respectively. The cumulative effects of body mass index and ‘a 3.5 kg load’ carried may not be sufficient to exhibit significant gait changes. First, the participants recruited for this study were a somewhat homogenous group of healthy individuals, with a majority of them being classified as ‘normal weight’. Second, a heavier load carried linearly leads to greater gait changes [27] and asymmetries in ground reaction forces and plantar pressures [12], while ‘a 3.5 kg load’ does not seem to produce such large, but only small effects. Based on the evidence, it is suggested that the safest and most biomechanically appropriate way to carry a load is by using a backpack, keeping the load close to the center of gravity [34]. Although we observed only trivial to small differences between ‘no load’ vs. ‘a 3.5 kg load’, there is still an implication of our findings in terms of re-positioning the items of the load. For example, the handgun can be moved to the lateral side of the thigh area to enable the arms to move swiftly during walking. We descriptively observed that the dominant arm often ‘freezes’ during gait, which increases movements on the opposite side of the body by increasing the lateral flexion of the trunk. In Croatia, the internal policy still dictates that police loads need to be attached around the hips, and future research on this topic are still warranted. Thus, strategies of re-designing police equipment and re-positioning it near the center of body should be implemented within the police system in order to minimize negative effects from the external load on the force and pressure distributions beneath the different foot regions. According to research by Quesada et al. [32], there is a physiological impact of load carriage on the human body. Carrying an additional load equivalent to 15% of the body weight results in a 5–6% increase in metabolic cost. In our own study, we found that a 3.5 kg load, which represents a relative value for our sample, may not significantly increase metabolic cost. However, it can lead to a more pronounced forward lean and distort gait patterns, as indicated by Bobet and Norman [31]. While a 3.5 kg load may not seem substantial enough to induce negative changes in gait, our study revealed that it can lead to increased asymmetries during the gait cycle. Load carriage influences on the anteroposterior and mediolateral planes of the foot, resulting in higher ground reaction forces and plantar pressures, which could lead to discomfort and pain during walking, as noted in previous studies [15,35,36]. Additionally, it may contribute to greater asymmetries between the left and right foot.

Practical implications of our findings can be useful in the field of practice among police officers, because even a small mass of police equipment can lead to a decrease in stability when walking in all planes. On the other hand, in addition to the basic equipment worn by police recruits, the mass of police equipment increases with the difficulty of the task, which can result in even more kinetic asymmetries of gait and increased forces and pressures under certain regions of the feet. One of the mechanisms of prevention of these conditions is the reorganization of police equipment during walking, with the aim of moving the pistol (which is normally carried on the hip) more towards the side of the upper leg, so that the hand on the side of the pistol can have a normal swing while walking. Also, in this way, the position of the torso would move more towards a neutral position (upright stance), which would directly lead to an equal distribution of forces and pressures under the feet on the ground. However, it is important to acknowledge the limitations of our study. The cross-sectional design restricts our ability to establish causal changes in asymmetries and limits the generalizability of our findings to police recruits. Furthermore, our focus on kinetic gait parameters means that we may have missed out on valuable insights provided by 3D kinematic and electromyography systems, which may be served in an inverse dynamic approach for testing torques within each joint. The absence of data pertaining to biological and physiological parameters, injury history and load-carrying techniques further restricts the practical implications of our findings. Finally, the fact that participants walked barefoot over the pressure platform could have impacted the observed gait patterns. Although the nature of the police work strictly dictates wearing appropriate shoes on duty, the methodology of the Zebris platform indicates that all measurements over the platform need to be barefoot-specific, since different types of shoes may mimic true values in ground reaction forces and plantar pressures by absorbing a significant amount of force within the shoe structure. Moving forward, it is essential for subsequent research to adopt a follow-up design and conduct comprehensive physiological and biomechanical analyses. Such studies should also consider load- and injury-related characteristics to mitigate the adverse effects of load carriage on gait.

## 5. Conclusions

Findings of this study indicate that ‘a 3.5 kg load’ significantly increases ground reaction force and plantar pressure gait asymmetries beneath the forefoot and midfoot regions, compared to a ‘no load’ condition. Such asymmetries may have hazardous effects on gait stability and an increased likelihood for musculoskeletal injuries, due to foot pain and discomfort. These negative changes may impact foot placement on the ground and increase an incidence for future stress fractures and deviated gait biomechanics in police recruits.

## Figures and Tables

**Table 1:** Basic descriptive statistics of the study participants at baseline.

Variables	Mean (SD)/N (%)	Min–Max	Range
Gender			
Men	609 (72.1%)		
Women	236 (27.9%)		
Age (years)	21.3 ± 2.1	18.7–24.7	6.0
Height (cm)	175.2 ± 14.3	164.3–190.8	26.5
Weight (kg)	74.4 ± 14.5	57.3–100.6	43.3
Body Mass Index (kg/m^2^)	24.3 ± 4.8	19.4–28.3	8.9

**Table 2:** Gait changes (mean ± SD) in ground reaction forces and plantar pressures beneath different foot regions.

Study Variables	Load Condition		*t*-Value	*p*-Value
	‘No Load’	‘a 3.5 kg Load’		
**Sex, N (%)**				
Men/Women	609 (72.1%)/236 (27.9%)	609 (72.1%)/236 (27.9%)	0.000	1.000
Age (years)	21.3 ± 2.1	21.3 ± 2.1	0.000	1.000
Body Mass Index (kg/m^2^)	24.3 ± 4.8	25.4 ± 4.5	−2.176	0.037
**Maximal Ground Reaction Forces**				
**Left Foot**				
Forefoot (N)	758.58 (130.70)	780.44 (135.94)	−3.351	< 0.001
Midfoot (N)	145.58 (71.58)	150.83 (78.34)	−2.083	0.037
Hindfoot (N)	513.65 (98.57)	524.38 (98.53)	−1.432	0.152
**Right Foot**				
Forefoot (N)	766.11 (304.00)	798.78 (336.76)	−1.497	0.135
Midfoot (N)	156.84 (79.10)	162.52 (76.32)	−2.227	0.026
Hindfoot (N)	500.23 (98.86)	508.53 (98.31)	−1.923	0.045
**Maximal Plantar Pressures**				
**Left Foot**				
Forefoot (N/cm^2^)	44.40 (9.80)	45.28 (9.76)	−1.857	0.049
Midfoot (N/cm^2^)	15.01 (7.54)	15.31 (7.60)	−1.088	0.277
Hindfoot (N/cm^2^)	33.05 (7.59)	33.69 (7.28)	−0.809	0.419
**Right Foot**				
Forefoot (N/cm^2^)	44.55 (10.07)	45.08 (9.91)	−1.900	0.046
Midfoot (N/cm^2^)	15.03 (6.52)	15.64 (6.62)	−1.855	0.049
Hindfoot (N/cm^2^)	31.89 (7.07)	32.46 (7.16)	−1.646	0.100
**Time Maximal Force, % of Stance Time**				
**Left Foot**				
Forefoot (%)	74.44 (2.44)	74.79 (2.13)	−3.101	0.002
Midfoot (%)	41.30 (9.62)	41.02 (9.82)	−2.538	0.011
Hindfoot (%)	18.47 (3.69)	18.88 (3.60)	0.570	0.569
**Right Foot**				
Forefoot (%)	74.16 (3.48)	74.52 (2.28)	−0.596	0.552
Midfoot (%)	39.70 (9.02)	40.00 (9.10)	−2.303	0.021
Hindfoot (%)	18.06 (3.74)	18.27 (4.05)	−1.102	0.271

**Table 3:** Differences in asymmetries between the left and right foot of the body in ‘no load’ vs. ‘a 3.5 kg load’ (mean ± SD).

Study Variables	Asymmetry		Mean Diff.	95% Mean Diff.	*p*-Value
Ground Reaction Forces *	‘No Load’	‘A 3.5 kg Load’			
Forefoot	0.000 (0.049)	0.014 (0.010)	−0.014	−0.021–0.006	<0.001
Midfoot	0.038 (0.192)	0.076 (0.201)	−0.038	−0.056–−0.019	<0.001
Hindfoot	−0.014 (0.058)	−0.025 (0.058)	0.011	0.005–0.017	<0.001
Plantar Pressures *					
Forefoot	0.001 (0.092)	0.000 (0.089)	0.001	−0.008–0.010	0.779
Midfoot	0.009 (0.171)	0.041 (0.172)	−0.032	−0.049–−0.016	<0.001
Hindfoot	−0.017 (0.085)	−0.019 (0.071)	0.002	−0.005–0.010	0.562
Time Maximal Force, % of Stance Time *					
Forefoot	−0.003 (0.036)	−0.002 (0.017)	−0.001	−0.003–0.002	0.674
Midfoot	−0.018 (0.105)	−0.011 (0.104)	−0.007	−0.017–0.003	0.143
Hindfoot	−0.013 (0.102)	−0.030 (0.101)	0.018	0.008–0.027	<0.001

* All models were adjusted for body mass index.

## Data Availability

The raw data supporting the conclusions of this article will be made available by the authors on request.
